# 99 Feasibility of Deep Learning-based Automatic Myofiber Size Measurement for Burn-induced Muscle Wasting and Its Reversal

**DOI:** 10.1093/jbcr/irac012.102

**Published:** 2022-03-23

**Authors:** Hiroyuki Morinaga, Yoh Sugawara, Jingyuan Chen, Jeevendra Martyn, Shingo Yasuhara

**Affiliations:** Kyorin University, Tokyo, Tokyo; Shriners Hospital for Children, Boston, Boston, Massachusetts; The First Affiliated Hospital of Sun Yat-Sen University, Guangzhou, Guangdong; Shriners Hospitals for Children, Boston, Massachusetts; Shriners Hospital for Children, Boston, Massachusetts

## Abstract

**Introduction:**

Patients with major burn injury (BI) often develop muscle wasting (MW) and mitochondrial dysfunctions (MD), which affect their prognosis. We have recently shown that auto/mitophagy response is defective in BI model and can be mitigate by trehalose treatment. Though auto/mitophagy is widely accepted as the quality control (QC) system of cellular components including mitochondria, the relationship among MD, auto/mitophagy response defect, and MW was unclear. Furthermore, to evaluate MW precisely by morphometric analyses was difficult, due to the ehavy workload of counting the size of muscle cross sectional area manually and analyzing the data. Thus, we have set up a streamline of whole section image capturing, analyzing with cutting edge deep learning-based method, processing it via image J-based program. Using this system, we have tested the efficacy of trehalose on mitigating MW in BI-treated mice.

**Methods:**

First, the effect of auto/mitophagy modulator on normalizing defective auto/mitiophagy maturation was confirmed by in vivo microscopy of tfLC3-expressing mice with BI (30% BSA) or sham-burn (SB) control. A mitophagy inducer, CCCP was injected to induce mitophagy, and the auto/mitophagosome maturation was monitored with or without trehalose treatment. In a separate experiment, tibialis muscles were harvested at post-burn day (PBD)-7, with or without trehalose treatment (2g/kg/day, i.p.), cryosectioned, and stained by anti-laminin antibody. The entire tissue cross-sectional microscopic images were captured, fed into a cellpose, and processed in ImageJ and Prizm for automatic calculation of the cross sectional area (CSA).

**Results:**

In vivo microscopic monitoring of auto/mitophagosome maturation revealed BI-induced maturation defect when treated by CCCP, which was rescued by trehalose treatment. Next, with MW analysis experiment, cross-sectional morphometric analysis of tibialis anterior myofibers showed a typical bi-phasic pattern of CSA distribution (large size population and small size population) in the control group. BI treatment showed a significant CSA decrease in both populations, which was effectively treated by trehalose. The average CSA was as follows (1843.0, 1245.3, 1683.9 for SB control, BI, BI+trehalose, respectively, in micron^2), and in accordance with manual counting measurement.

**Conclusions:**

Normalizing defective auto/mitophagy response was shown an effective therapeutic approach to mitigate BI-induced MW. Deep learning-based size counting method is a feasible technique for a systematic MW analysis. Note that in our data, trehalose does not function in increasing the basal level of autophagy, but it mitigates the defective response of auto/mitophagy to the auto/mitophagic stimulation, by normalizing the maturation process.

